# Improved diagnostic management of children with acute infections following the introduction of point-of-care C-reactive protein testing and general practitioner education in Latvia: a *post hoc* analyses of a randomised controlled intervention study

**DOI:** 10.1080/02813432.2025.2571927

**Published:** 2025-10-29

**Authors:** Zane Likopa, Anda Kivite-Urtane, Ieva Strele, Jana Pavare

**Affiliations:** a Children’s Clinical University Hospital, Riga, Latvia; b Riga Stradins University, Riga, Latvia; c Institute of Public Health, Riga Stradins University, Riga, Latvia; d Institute of Occupational Safety and Environmental Health, Riga Stradins University, Riga, Latvia; e Steno Diabetes Center Aarhus, Aarhus N, Denmark

**Keywords:** Acute infections, children, point-of-care testing, antibiotic prescription, primary care

## Abstract

**Objective:**

In order to reduce unnecessary antibiotic prescribing, diagnostic processes require improvement for children in primary care.

**Design:**

*Post hoc* analyses of randomised controlled intervention study.

**Setting:**

Eighty general practitioner (GP) practices throughout Latvia.

**Intervention:**

In the first study period, one GP group received combined interventions (access to CRP POCT and GP education), while the second GP group continued usual care (control group). In the second study period, the GP groups were switched – previous control group received combined intervention, but previous intervention group re-established usual care, but the long-term education effect was evaluated in this group.

**Subjects:**

Children with acute infections consulted by a GP.

**Main outcome:**

Impact of combined intervention and long-term education on testing level (CRP, full blood count, Strep A test, influenza test, urinalysis and X-ray) before antibiotic prescribing. Patient- and GPs- related predictors (including practice location and access to laboratory services) of diagnostic testing were also analysed. Secondary outcome was antibiotic prescribing according to the test results.

**Results:**

Diagnostic testing was significantly increased in the combined intervention group versus the usual care group (aOR 11.1, 95% CI 8.0–15.3); however, it was decreased in the long-term education group (26.4%) (aOR 0.5, 95% CI 0.3–0.8). Rural practices and a longer expected time of laboratory results were associated with a more pronounced increase in diagnostic testing in the combined intervention group (aOR 37.6, 95% CI 17.9–79.0; aOR 23.2, 95% CI 14.1–38.0, respectively). It was found that a low CRP value, negative Strep A test or normal X-ray often did not convince GPs to withhold antibiotics.

**Conclusion:**

The availability of CRP POCT and GP education results in a much higher level of diagnostic testing prior to antibiotic prescribing, especially in rural regions. Further improvements in more rational testing and the interpretation of results to guide appropriate antibiotic prescribing are essential.

**Trial registration:** ISRCTN registry - ISRCTN34931655

## Introduction

The World Health Organisation has declared increasing antimicrobial resistance to be a major threat to global health [1]. Primary care accounts for 80–90% of all antibiotic prescriptions [2,3], with around one-third thought to be inappropriate [1,2,4]. Acute infections are the leading cause of general practitioner (GP) consultations among paediatric patients [5]. Rational decisions about antibiotic prescribing are based on a proper diagnosis [6]. Typically, children are suffering from self-limiting viral infections, in which case antibiotic treatment is not beneficial. However, patients frequently present with non-specific symptoms and bacterial infections that can be difficult to differentiate based solely on clinical symptoms [2,7].

GPs often prescribe antibiotics when they are unsure about the clinical outcome and are fearful of missing a serious bacterial infection. Additionally, GPs often make decisions under pressure due to parental insistence on antibiotics being prescribed. Furthermore, heavy workloads and limited time for consultations also increase the likelihood of antibiotics being prescribed [2,4,8]. Having said that, parental concerns and requests for medical evaluation of their child may be misinterpreted as requests for antibacterial treatment [8]. Previous studies have shown that point-of-care test (POCT) usage can enhance the communication with parents, reduce clinician uncertainty for physicians and improve targeted treatments [9,10]. POCTs are easy to perform during consultations and, by providing immediate results, are especially appropriate for primary care [11]. Moreover, they can improve patient flow within busy schedules and monitoring [9]. Considerable variation in POCT usage has been reported in European countries, ranging from 0% in Croatia and Moldova up to 65% in Norway and Denmark [12].

C-reactive protein (CRP) is one of the most frequently studied markers of an acute inflammatory response that can help differentiate serious bacterial infections and non-bacterial infections and thus reduce antibiotic prescribing [13]. In the last few years, measurement of the level of CRP has been increasingly adopted as a POCT in primary care [11]. Previous systematic reviews have shown that CRP POCT significantly reduced antibiotic prescribing for respiratory infections for adults [14–16] and testing is included in lower respiratory infection guidelines [17]. Regarding child age patients, CRP POCT likely reduces unnecessary antibiotic prescribing for children with respiratory infections. The effect appears to be more pronounced in lower-middle-income countries when guidance of on interpretation of CRP POCT is provided. However, evidence from high-income countries, where baseline antibiotic prescribing rates are already lower, remains inconclusive [16,18,19]. The latest Cochrane review also concluded that future research should focus on the paediatric population [16]. Since CRP POCT has the potential not only to reduce antibiotic prescribing and diagnostic uncertainty, but also to support communication with parents and patients when antibiotic are withheld, establishing best practices for its use in pediatric primary care settings is essential [1].

In the original trial, we have shown that the availability of CRP POCT and educational training for GPs together did not reduce antibiotic prescribing for children with acute infections, but regional variations in antibiotic prescribing habits exist and the implemented interventions had an effect in rural areas [20,21].

The present *post hoc* analyses assessed how a combined intervention and long-term education influenced diagnostic testing prior to antibiotic use, along with patient- and GP-related factors affecting testing and whether the antibiotic prescription was affected by the test results.

## Materials and methods

We conducted a *post hoc* analyses of a randomised controlled intervention study carried out in general practice settings in Latvia between November 2019 and April 2021. The study methodology and the main results of the effects of CRP POCT and GP education on antibiotic prescribing have been published previously [20,21].

### 1. Study design

**Figure 1.**
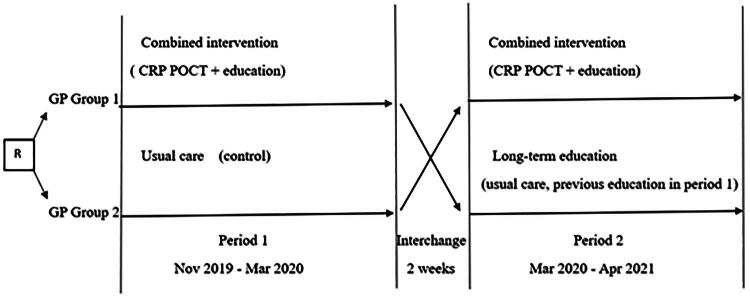
Study overview. R: randomisation GP – general practitioner CRP POCT – C-reactive protein point-of-care test.

The study consisted of two periods (Figure 1). In the first study period, GPs who were randomly enrolled in the control group continued usual care with no specific recommendations regarding antibiotic prescribing. GPs assigned to the combined intervention group attended an educational session and received a CRP POCT device for use during the first study period. After a three-month period, GPs were interchanged: the control group received the combined intervention and the combined intervention group in the first study period re-established usual care. This latter group (long-term education group) was analysed separately as the education the GPs received earlier in the first study period could have an impact on diagnostic habits and antibiotic prescribing.

### 2. Participants

GPs were selected from all five of the planning regions in Latvia, with both urban and rural practices being represented. In Latvia, GP is recognised as a specialty, equivalent to other medical specialties. Becoming a GP requires three years of specialist training with recertification every 5 years. GPs manage patients of all ages, including children, and are typically self-employed, often working in independently located practices. The participating GPs had varying levels of work experience, ranging from 1 to 52 years, and the number of paediatric patients per practice varied considerably, from 48 to 1,843 children. Eighty GPs were randomly assigned to either the usual care or combined intervention group using random numbers generated by the MS Excel Random Number function with stratification according to practice location. Details of the recruitment procedure can be found in previous publications [20,21].

GPs registered the data of children aged one month to 17 years who attended their practice for a face-to-face GP consultation and presented with an acute illness episode of a maximum of five days. Patients in the reconvalescent stage or who had received antibiotics during this illness episode prior to the practice visit were excluded.

### 3. Intervention

The intervention consisted of a combination of CRP POCT and GP education.

### 3.1. Educational session

GPs were asked to participate in one four-hour training seminar, followed by educational materials in video and printed format. The educational session was focused on new recommendations for the management of respiratory infections and fever in children that were introduced in Latvia. The key topics were:

- child with fever – evaluation, precautionary level system and management,
- child with upper and lower respiratory infection – evaluation and management,
- principles of antibiotic resistance and safer prescribing of antibiotics

### 3.2. CRP POCT

GPs received a CRP POCT device (Orion Diagnostica QuikRead go CRP POCT system) for use during the duration of the study and were individually instructed in performing CRP POCT by the manufacturers during a face-to-face meeting. Furthermore, they received ongoing support from the manufacturers throughout the study. According to new recommendations presented in educational session, CRP testing was recommended for children with fever and respiratory infections when a serious bacterial infection was suspected based on clinical assessment. Meanwhile, GPs were allowed to use the test for any patient in whom they considered it could support antibiotic prescribing decisions following clinical assessment. Specific thresholds were not provided for decision-making, as currently, there are no standardized threshold values for this age group defined for the interpretation of the test results [22,23].

### 4. Data collection and outcomes

GPs recorded data in anonymized forms about each patient’s demographic data, duration of illness, diagnosis based on a pre-defined list (upper respiratory infections (common cold, rhinosinusitis, otitis, pharyngitis, tonsillitis, stomatitis, laryngitis), lower respiratory infections (bronchitis, bronchiolitis, pneumonia), gastrointestinal infections, urinary tract infections, skin and soft tissue infections and joint and bone infections), planned actions – investigations performed (POCT, blood tests, X-ray, urinalysis), decision on antibiotic treatment and referral to hospital or ambulatory care.

The primary outcome was the level of diagnostic testing prior to antibiotic prescribing, (CRP, full blood count, Strep A test, influenza test, urinalysis, and X-ray), following the combined intervention and long-term education. Patient- and GP-related predictors of diagnostic testing, including practice location and access to laboratory services, were also evaluated. The secondary outcome was antibiotic prescribing in accordance with the results of these tests.

### 5. Statistical analysis

Descriptive statistics, such as proportions for categorical variables, means and medians (with interquartile range (IQR)) for continuous variables, were calculated. The conformity of continuous variables to a normal distribution was tested using the Kolmogorov–Smirnov test. The Chi-square test was used to determine the statistical significance of differences in the proportions of the dependent variable between subgroups of the independent variable. To evaluate the statistical significance of differences in stratified median values, the Mann–Whitney or the Kruskal–Wallis test was used.

A generalised linear mixed model with a binomial distribution (logistic mixed effect model) was used to assess the association between the study intervention and diagnostic testing as the outcome, adjusted for other factors. Odds ratios of the use of diagnostic tests and 95% confidence intervals (CI) were calculated. To account for clustered observations within GP practices, all models included GPs’ identification numbers as random intercepts. Both GP-related (age, work experience, urbanicity of a GP practice location and the number of paediatric patients) and patient-related (age, sex, site of infection, duration of symptoms, presence of chronic diseases and vaccination status) factors, as well as the study intervention, were included as fixed effects. Due to the collinearity between GP age and work experience, the multiple regression model included either age or work experience. Besides, the study intervention group had a significant interaction (*p* < 0.001) with the location of a GP practice and the expected time of laboratory results. Therefore, separate models were fitted by the level of urbanicity of the GP practice location and whether the laboratory results were expected on the same day or later. Results were considered statistically significant at *p* < 0.05. Data were processed using IBM SPSS Statistics (Statistical Package for the Social Sciences) Version 26.0.

## Results

### 1. GP and patient flow

Patients with acute infections were registered between November 2019 and April 2021. Initially, 80 GPs participated in the study; however, five declined to participate further after randomisation and two more refused to participate in the second study period. The mean number of patients enrolled was 44.2 per GP.

Of 3,801 patients enrolled, 484 did not fulfil the inclusion criteria or did not have complete data and were excluded. The number of patients in each study group was: 1,784 in the combined intervention group, 886 in the usual care group and 647 in the long-term education group. Patient and GP flow has been described previously [20].

### 2. Patient characteristics according to study group

Of the 3,317 participants, 49.3% were boys. The median age was 4.0 years (IQR 2.0–8.0) and 8.2% had any comorbidity, with bronchial asthma being the most common (85.1%). The median duration of symptoms was three days (IQR 2.0–4.0). The patient characteristics in the study groups have been described previously [20]. According to the diagnoses recorded by GPs, respiratory infections were the most common presentation in all three study groups (Table 1). There was only a very small number of patients with skin and soft tissue infections and bone and joint infections included in the study.

**Table 1.** Diagnoses recorded by GPs according to the three study groups.

Infection location	Diagnoses subgroups*	Combined intervention group n (%)	Usual care group n (%)	Long-term education group n (%)
Upper respiratory infections	Otitis	116 (6.5)	62 (7.0)	45 (7.0)
	Pharyngotonsillitis	398 (22.3)	199 (22.5)	140 (21.6)
	Rhinosinusitis	112 (6.3)	37 (4.2)	95 (14.7)
	Common cold	750 (42.0)	377 (42.6)	215 (33.2)
Lower respiratory infections	Bronchitis	285 (16.0)	155 (17.5)	94 (14.5)
	Pneumonia	52 (2.9)	25 (2.8)	14 (2.2)
Gastrointestinal infections		41 (2.3)	19 (2.1)	13 (2.0)
Urinary tract infections		20 (1.1)	10 (1.1)	19 (2.9)
Skin and soft tissue infections		8 (0.5)	1 (0.1)	11 (1.7)
Bone and joint infections		2 (0.1)	1 (0.1)	1 (0.2)
Total (n)		1784	886	647

*Diagnoses subgroups were established only for the largest patient groups, i.e. upper and lower respiratory infections.

### 3. Diagnostic management prior to antibiotic prescribing

Overall, 52.5% (*n* = 1,742) of the study population received a diagnostic test(s) before antibiotic prescribing. Measurement of the level of CRP was the most frequently performed test (42.5%), whereas all other tests were rarely performed (Strep A, 6.6%; full blood count, 5.6%; urinalysis, 4.7%; influenza, 3.7%; X-ray, 3.0%).

A multilevel analysis was performed on different patient- and GP-related factors associated with diagnostic testing (Table 2). Crude analyses on patient-related factors revealed that older age of children was associated with higher testing: compared to the 0–4-year-old children, those at age 5–9, age 10–14, and age 15–17 had received diagnostic tests 1.3 (*p* = 0.003), 1.5 (*p* = 0.001), and 2.9 (*p* < 0.001) times more, respectively. Longer duration of symptoms was also associated with a higher usage of diagnostic tests: ORs of testing gradually increased from 2.3 for a 2-day length of symptoms to 5.0 for a length of 5 days compared to 1-day duration of symptoms (all *p* < 0.001). Besides, compared to the upper respiratory infections, urinary tract infections and lower respiratory infections had higher odds of diagnostic testing: 8.0 (*p* < 0.001) and 1.4 (*p* = 0.002), respectively. At the same time, among the GP-related factors, the only factor having a statistically significant association with diagnostic testing was the study intervention. Thus, belonging to the combined intervention group (provided with the CRP POCT equipment) increased odds of testing 11.5 times compared to the control group (*p* < 0.001), but belonging to the long-education group (without the testing equipment) was associated with 40% lower odds of testing compared to the control group. Following adjustment, all the significant factors from the crude analyses remained statistically significant, except for age groups 5–9 and 10–14 years of children. Similarly, both the strength and statistical significance of the association between the study intervention and diagnostic testing remained almost unchanged.

**Table 2.** Patient- and GP-related predictors of diagnostic test usage.

Characteristics	Tested patients n (%)	Crude OR (95% CI)	*p*	Adjusted OR* (95% CI)	*p*
**Patient-related factors**					
Age (years)					
15–17	119 (71.3)	**2.9 (1.9–4.3)**	**<0.001**	**2.2 (1.4–3.7)**	**0.002**
10–14	242 (56.8)	**1.5 (1.2–2.0)**		1.3 (1.0–1.8)	0.080
5–9	548 (54.5)	**1.3 (1.1–1.5)**	**0.001**	1.2 (1.0–1.5)	0.131
0–4	812 (48.2)	1	**0.003**	1	
Sex					
Girls	844 (52.0)	1.0 (0.8–1.1)	0.655	0.9 (0.7–1.0)	0.095
Boys	886 (53.0)	1		1	
Duration of symptoms (days)					
1	49 (25.9)	1		1	
2	468 (46.4)	**2.3 (1.6–3.5)**	**<0.001**	**1.7 (1.0–2.6)**	**0.032**
3	616 (54.9)	**3.1 (2.1–4.7)**	**<0.001**	**2.2 (1.4–3.4)**	**0.001** **<0.001**
4	373 (59.6)	**4.2 (2.8–6.4)**	**<0.001**	**2.9 (1.8–4.7)**	
5	234 (64.1)	**5.0 (3.2–7.8)**	**<0.001**	**2.6 (1.5–4.5)**	**<0.001**
Location of infection					
Upper RTI	1260 (49.5)	1		1	
Lower RTI	382 (61.1)	**1.4 (1.1–1.8)**	**0.002**	**1.4 (1.1–1.9)**	**0.01**
Gastrointestinal	48 (65.8)	1.5 (0.9–2.6)	0.147	1.8 (0.9–3.5)	0.101
UTI	44 (89.8)	**8.0 (3.0–21.3)**	**<0.001**	**28.2 (9.0–87.7)**	**<0.001**
Skin, soft tissue, bone and joint	8 (33.3)	0.4 (0.2–1.2)	0.099	0.8 (0.3–2.6)	0.751
Comorbidities					
Yes	133 (49.1)	0.9 (0.7–1.2)	0.374	0.9 (0.6–1.3)	0.532
No	1609 (52.9)	1		1	
Vaccination					
Complete	1598 (52.9)	1		1	
Incomplete	99 (52.4)	0.7 (0.5–1.1)	0.098	0.8 (0.5–1.20)	0.266
No	15 (45.5)	0.9 (0.4–2.0)	0.792	1.2 (0.4–3.5)	0.729
**GP-related factors**					
Age (years)					
30–40	291 (43.8)	1		1	
41–50	385 (52.2)	1.6 (0.5–4.8)	0.421	1.1 (0.3–4.2)	0.897
51–60	550 (53.3)	1.5 (0.6–4.2)	0.418	1.0 (0.3–3.1)	0.943
61+	516 (58.4)	2.5 (0.9–7.5)	0.091	2.0 (0.6–7.0)	0.295
Work experience (years)					
≤5	192 (53.9)	1		1	
6–10	72 (36.7)	0.4 (0.1–2.1)	0.256	0.4 (0.0–3.5)	0.402
11–20	211 (39.2)	0.5 (0.1–2.2)	0.383	0.3 (0.1–1.8)	0.203
21+	1267 (56.9)	1.3 (0.4–3.9)	0.689	0.8 (0.2–3.0)	0.693
Location of practice					
Rural areas	634 (54.1)	1.1 (0.5–2.4)	0.877	1.2 (0.4–3.4)	0.715
Regional cities	369 (52.8)	1.5 (0.6–3.6)	0.426	1.4 (0.5–4.1)	0.532
Capital city	739 (51.1)	1		1	
Number of paediatric patients in practice					
≤500	226 (58.1)	2.1 (0.6–7.9)	0.246	4.6 (1.0–21.7)	0.057
501–1000	673 (54.1)	1.1 (0.5–2.4)	0.771	1.1 (0.4–2.9)	0.808
1001+	843 (50.1)	1		1	
Study group					
Combined intervention	1294 (72.5)	**11.5 (8.5–15.8)**	**<0.001**	**11.1 (8.0–15.3)**	**<0.001**
Long-term education	171 (26.4)	**0.6 (0.4–0.9)**	**0.008**	**0.5 (0.3–0.8)**	**0.006**
Usual care	277 (31.3)	1		1	

The data presented are results of logistic mixed effects models with GP practices as random effects.

*Adjusted for all independent variables in the table, except for GP age due to collinearity with work experience (for GP age, variable adjustment was carried out for all the variables except work experience).

RTI: respiratory tract infection; UTI: urinary tract infection; GP – general practitioner.

**Figure 2.**
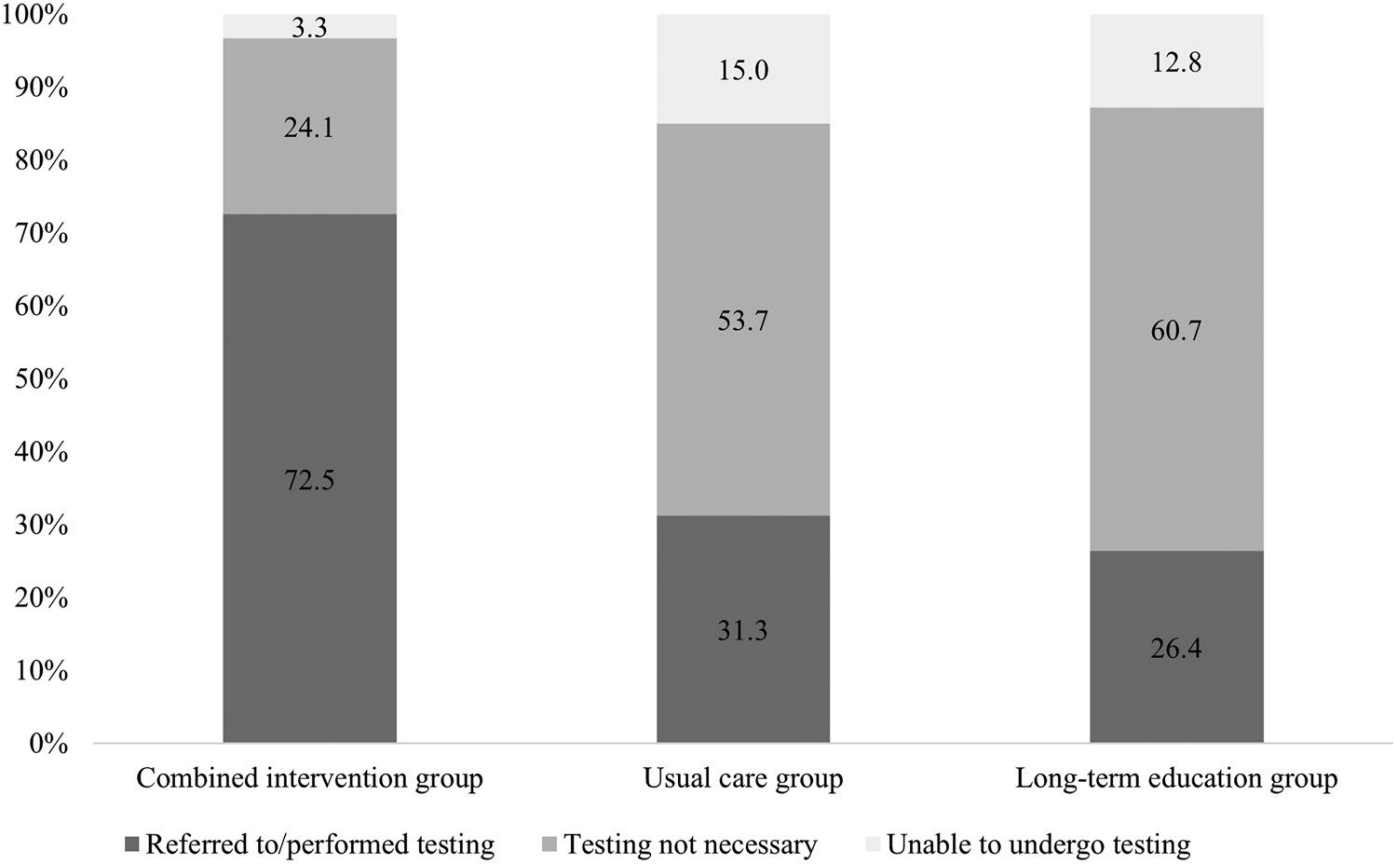
Comparison of availability and use of diagnostic tests in the three study groups.

Overall, the diagnostic testing level was highly variable among the three study groups (Figure 2); high (72.5%) in the combined intervention group when CRP POCT was available and significantly lower in the usual care (31.3%) and long-term education (26.4%) groups (*p* < 0.001). In addition, 15.0% of cases in the usual care group and 12.8% of cases in the long-term education group were deemed by GPs to warrant testing but were unable to undergo testing before antibiotic prescribing due to the time delay of laboratory results or lack of POCT in the practice. This was compared to only 3.3% of cases in the combined intervention group (*p* < 0.001).

Due to a significant interaction (*p* < 0.001), the effect of the study intervention varied according to the urbanicity level of GP practice and the expected time of laboratory results. Specifically, in the combined intervention group, diagnostic testing increased more than 30 times in rural practices (aOR 37.6, *p* < 0.001) and regional city practices (aOR 31.6, *p* < 0.001) compared to only five times in capital city practices (aOR 5.0, *p* < 0.001) where accessibility to laboratories is considerably better (Table 3). Similar results were observed in the combined intervention group regarding the expected time of laboratory results, where practices waiting more than one working day for results were associated with a more pronounced increase in diagnostic testing (aOR 23.2, *p* < 0.001) compared to practices with results during the same working day (aOR 5.6, *p* < 0.001).

**Table 3.** Effect of interventions (CRP POCT and GP education) on diagnostic testing according to practice location and expected time of laboratory results.

	Combined intervention		Long-term education		Combined intervention		Long-term education	
	Crude analyses				Adjusted analyses*			
Characteristics	OR (95% CI)	p	OR (95% CI)	p	aOR (95% CI)	p	aOR (95% CI)	p
Expected time of laboratory results								
During working day	**5.5 (3.6–8.4)**	**<0.001**	**0.3 (0.2–0.6)**	**<0.001**	**5.6 (3.6–8.9)**	**<0.001**	**0.3 (0.2–0.6)**	**<0.001**
>1 working day	**24.0 (15.1–38.4)**	**<0.001**	0.8 (0.4–1.7)	0.594	**23.2 (14.1–38.0)**	**<0.001**	0.7 (0.3–1.5)	0.385
Practice location								
Rural	**35.5 (17.4–72.3)**	**<0.001**	**0.2 (0.1–0.6)**	**0.002**	**37.6 (17.9–79.0)**	**<0.001**	**0.1 (0.0–0.4)**	**<0.001**
Regional cities	**22.1 (10.6–45.7)**	**<0.001**	**2.6 (1.0–6.7)**	**0.048**	**31.6 (13.8–72.1)**	**<0.001**	**3.1 (1.1–9.3)**	**0.041**
Capital city	**5.4 (3.6–8.1)**	**<0.001**	0.9 (0.5–1.5)	0.59	**5.0 (3.3–7.5)**	**<0.001**	0.9 (0.5–1.6)	0.675

The data presented are results of logistic mixed effects models with GP practices as random effects. Results show the odds ratio of diagnostic testing in intervention groups (combined intervention and long-term education) versus the usual care group.

*Adjusted for patient-related factors (age, sex, duration of symptoms, location of infection, vaccination, comorbidities) and GP-related factors (work experience, number of paediatric patients in practice).

Use of diagnostic tests also varied widely across different diagnoses (Table 4). CRP testing was more frequently performed in the combined intervention group compared to the other study groups for all diagnoses except lower respiratory infections and gastrointestinal infections, where the difference was not statistically significant among the groups. Overall, more than half of patients with a respiratory infection underwent CRP testing in the combined intervention group.

In all the study groups, Strep A test was not heavily utilised for acute pharyngotonsillitis (12.9–29.1%). Instead, in the combined intervention group, CRP POCT was used for most of the pharyngotonsillitis patients (70.6%, *n* = 282). Similarly, CRP POCT was widely used for lower respiratory infections in the combined intervention group (79.3% (*n* = 226) for bronchitis, 69.2% (*n* = 36) for pneumonia), whereas X-ray was the predominant test for pneumonia patients in the usual care group (56.0%, *n* = 14). Urinalysis usage was similar for urinary tract infections in all the study groups; however, CRP POCT was utilised more often for these patients in the combined intervention group (70.0%, *n* = 12).

**Table 4.** Heatmap of diagnostic test usage (% of patients) prior to antibiotic prescribing according to diagnoses across the three study groups.

Diagnostic test		Combined intervention	Long-term education	Usual care		Combined intervention	Long-term education	Usual care
Strep A	**Otitis**	0.9	0	11.3	**Pneumonia**	0	0	0
CRP		52.6	6.7	9.7		69.2	21.4	44.0
FBC		0	6.7	4.8		3.8	28.6	32.0
Influenza		1.7	0	3.2		9.6	0	8.0
X-ray		0	0	0		28.8	21.4	56.0
Urinalysis		1.7	0	6.5		5.8	14.3	8.0
Strep A	**Pharyngotonsillitis**	19.3	12.9	29.1	**GI infections**	0	0	0
CRP		70.6	18.6	5.0		75.6	38.5	5.3
FBC		2.3	15.7	5.0		7.3	23.1	5.3
Influenza		1.5	0	8.5		2.4	0	0
X-ray		0	0.7	0		0	0	0
Urinalysis		2.3	5.7	3.0		12.2	30.8	31.6
Strep A	**Rhinosinusitis**	3.6	1.1	5.4	**UTI**	0	0	10.0
CRP		55.4	5.3	2.7		70.0	15.8	30.0
FBC		1.8	2.1	2.7		0	21.1	10.0
Influenza		0.9	0	8.1		0	0	10.0
X-ray		0.9	0	0		0	0	0
Urinalysis		4.5	0	5.4		45.0	78.9	80.0
Strep A	**Common cold**	3.3	0	1.3	**Skin and soft tissue**	12.5	0	0
CRP		69.3	16.7	6.1		12.5	0	0
FBC		1.6	15.8	5.3		12.5	0	0
Influenza		3.7	6.0	6.9		0	0	0
X-ray		1.7	2.3	0.5		0	0	0
Urinalysis		3.2	0.5	3.7		0	0	0
Strep A	**Bronchitis**	4.9	1.1	3.9	**Bone and joint**	0	100	0
CRP		79.3	16.0	14.2		50.0	0	100
FBC		2.8	14.9	11.6		0	0	100
Influenza		3.2	0	3.9		0	0	0
X-ray		7.0	13.8	7.7		0	0	0
Urinalysis		4.6	2.1	8.4		0	0	0

Red shades indicate higher proportion (%) of patients tested and green shades lower proportion.

FBC: full blood count; GI: gastrointestinal; UTI: urinary tract infection.

### 4. Relationship between diagnostic testing and antibiotic prescribing

**Figure 3.**
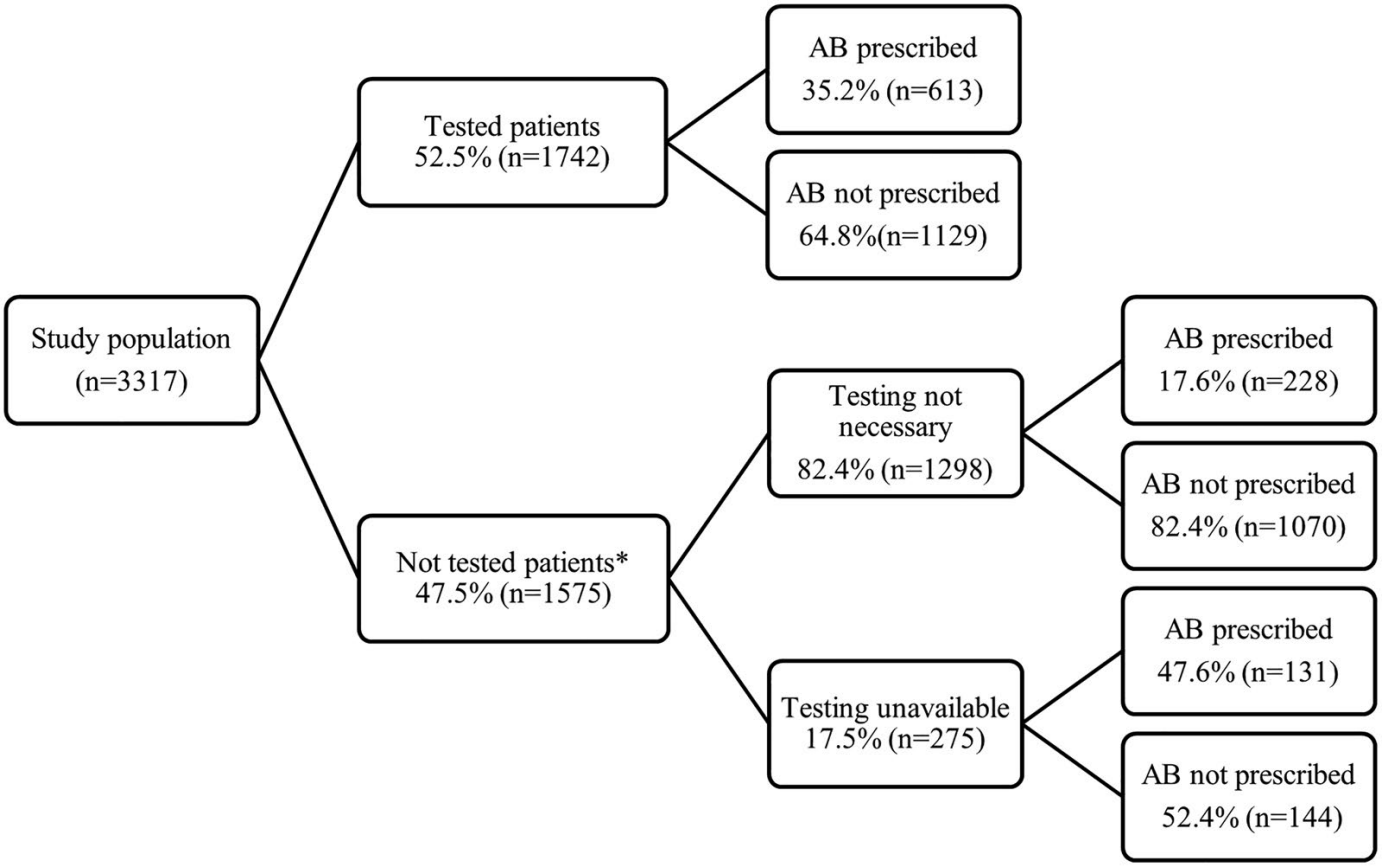
Proportion of patients receiving an antibiotic prescription according to diagnostic testing. *Two of patients not tested had no information whether tests were unavailable or not necessary. AB: antibiotics.

For 22.8% (*n* = 359) of patients, antibiotics were prescribed without diagnostic tests (CRP, full blood count, Strep A test, influenza test, urinalysis and X-ray) being performed (Figure 3). Almost half of patients (47.6%, *n* = 131) for which GPs deemed a diagnostic test(s) was necessary but unavailable at the time of consultation, received an antibiotic prescription. In contrast, a lower proportion of patients received an antibiotic prescription when a diagnostic test(s) was performed prior to making a decision (35.2%, *n* = 613). This was also the case when GPs thought diagnostic testing was not necessary (17.6%, *n* = 228). A statistically significant difference was found between all group pairs (*p* < 0.001).

**Figure 4.**
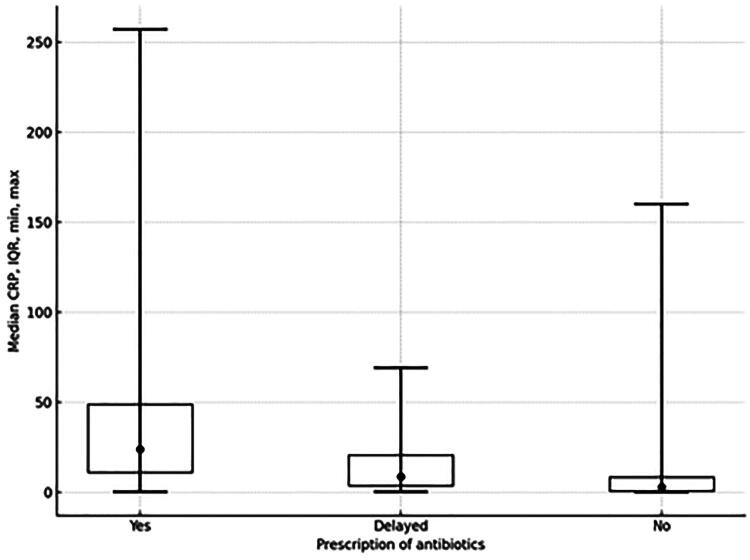
Median CRP boxplot for patients with, without, and with a delayed antibiotic prescription.

For most of the tested patients (78.3%), their CRP level was equal to or less than 20 mg/L. For 14.6% it was 20.01 to 50 mg/L, for 5.0% it was 50.01 to 99.99 mg/L and for only 2.1% it was equal to or greater than 100 mg/L. Median CRP was significantly higher for patients with an antibiotic prescription (24.0 mg/L (IQR 11.0–49.0)) versus a delayed antibiotic prescription (8.6 mg/L (IQR 3.7–20.8)) and versus no antibiotic prescription (2.9 mg/L (IQR 0.8–8.4) (*p* < 0.001 among all groups; Figure 4).

**Figure 5.**
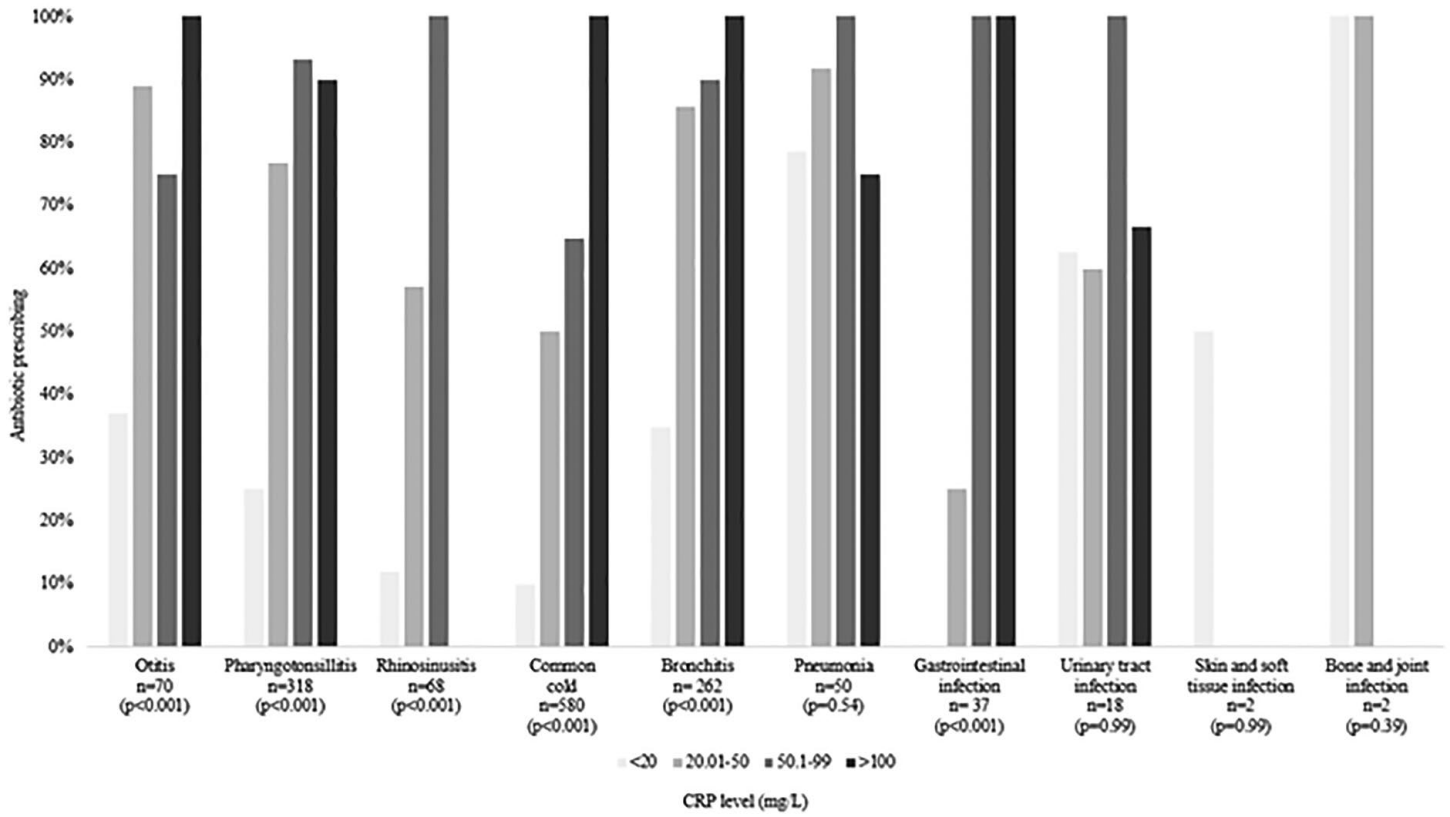
Proportion of patients receiving an antibiotic prescription according to CRP level for different diagnoses.

In general, antibiotic prescribing increased with increasing CRP level for all diagnoses (Figure 5). The exceptions were pneumonia and urinary tract infections, where the difference between antibiotic prescribing for patients with lower versus higher CRP levels was not statistically significant.

For patients with pharyngotonsillitis without a Strep A test, the antibiotic prescribing rate was 31.9% (*n* = 187). Most of the patients with a positive Strep A test received an antibiotic prescription (97.2%, *n* = 70); however, 40.7% (*n* = 33) of patients with a negative Strep A test also received an antibiotic prescription.

For the most part, patients with a positive influenza test (90.9%, *n* = 50) did not receive an antibiotic prescription.

If patients with a lower respiratory infection had a chest X-ray with no pathological changes, 50.0% of them still received an antibiotic prescription.

## Discussion

### Main findings

This study evaluated the diagnostic management of children with acute infections in general practice in Latvia (variations in ordering tests, factors associated with testing and interpretation of test results and subsequent antibiotic-prescribing decisions) and assessed whether CRP POCT implementation and GP education altered diagnostic habits.

We found that CRP POCT availability and GP education significantly increased diagnostic testing prior to antibiotic prescribing for all acute infections and reduced the number of cases where GPs were unable to perform the necessary diagnostic test(s), which was the group with the highest level of antibiotic prescribing. A more pronounced increase in diagnostic testing was observed with regard to practices located in rural areas and regional cities and also practices with a poorer accessibility to laboratories.

Regarding patient-related factors, age 15 to 17 years, longer duration of symptoms, and urinary tract and lower respiratory tract as location of infection showed an association with diagnostic testing. As for GP-related factors, by far the strongest association with diagnostic test usage was the study group to which the GP belonged.

Measurement of the level of CRP was the test most often performed by GPs in the study population. It was observed that CRP testing was lower in the usual care and long-term education groups where patient samples had to be sent to external laboratories.

Low CRP values of less than 20 mg/L, for which it is strongly advised to avoid antibiotics if clinical assessment rules out a severe infection [1], negative Strep A test for pharyngotonsillitis and normal X-ray for lower respiratory infections did not convince GPs to withhold antibiotics.

## Strengths and limitations

The data were prospective and collected from GPs covering different urban and rural practices, thus giving detailed insight into primary care in Latvia. As CRP POCT was not available in GP practices prior to commencement of this study, we were given the valuable opportunity to evaluate potential changes in diagnostic habits resulting from the introduction of this informative test in daily practice.

The data presented here were based only on the diagnoses established by the GPs during the patient’s visit; we do not have data on what clinical signs GPs based on their decision. Also, we focused on immediate antibiotic prescriptions given to the patient during their GP visit; we do not have data on further prescribing, outcome or hospitalization as they were not goals of the study. Although we reached the required sample size of 571 patients in each study group, several diagnoses, such as urinary tract infections, gastrointestinal infections, skin and soft tissue infections and bone and joint infections, were represented by a low number of children. Consequently, the results were mainly focused on respiratory infections.

We chose not to guide GPs with strict decision rules based on CRP levels, as safe cut-off values for children had not been well established at the time of the study. This might also have reduced the potential effect of the intervention.

The COVID-19 pandemic began during the study. Due to resultant social-distancing restrictions, the circulation of all respiratory viruses was lower. Thus, we had to extend the length of the study in order to reach the required sample size.

## Comparison with existing literature

To optimise correct antibiotic usage, it is essential to improve diagnostic processes [24]. As antibiotic overprescribing is often due to GPs being uncertain of the clinical outcome of an infection episode [8], one of the strategies to reduce unnecessary antibiotic prescribing in primary care is the use of POCT [18]. At present, there is no test available that is able to determine whether an infection is caused by a virus or bacterium. Therefore, testing should be strictly consistent with clinical assessment as overtesting could also lead to antibiotic overtreatment [16,25,26]. A wide variation in the diagnostic management of febrile children in hospital emergency departments has been reported across Europe [27]; however, only a few studies have been conducted in primary care. Moreover, to the best of our knowledge, the effect of CRP POCT implementation in clinical practice in combination with GP education on diagnostic habits has not been evaluated.

Our initial work found that the implementation of CRP POCT in general practice combined with GP education did not lead to a reduction in antibiotic prescribing. Nevertheless, regional variations in antibiotic prescribing were observed, with the combined intervention demonstrating an effect in rural areas [20,21]. In the post-hoc analysis presented here, we investigated the impact of the interventions on GPs diagnostic behaviour. We observed that CRP POCT implementation in general practice together with GP education significantly increased overall diagnostic testing prior to antibiotic prescribing. CRP POCT availability in GP practices also reduced the number of cases where GPs were unable to perform the necessary diagnostic test(s), which was the group with the highest level of antibiotic prescribing. A more pronounced increase in diagnostic testing was observed regarding practices with poor accessibility to laboratories, demonstrating that the introduction of POCT in rural regions is essential. In accordance, a previous study has shown that healthcare professionals who were able to access test results within 1–2 h from central laboratories found POCT less beneficial [9]. In some GP practices in our study, the turnaround time for CRP test results from laboratories could be up to two working days, which is far too long when making a treatment decision for an acute illness.

We found that older children were more likely to receive a diagnostic test. Although this finding is in conflict with data from a previous study where younger age was associated with testing [27], it is in line with a study from Denmark that also reported a higher level of testing for teenagers [28]. A higher level of testing was also associated with lower respiratory infections and urinary tract infections.

In the study population, CRP measurement was the most frequently used test for children with acute infections for all diagnoses. The use of this test significantly increased in the combined intervention group, where GPs had access to it as a POCT. CRP is a commonly used marker to detect inflammatory processes and guide antibiotic prescribing [28,29]. However, in primary care, this marker has limited usefulness if it has to be measured in central laboratories as there is a concomitant delay in receiving results. As a POCT in GP practices, it offers several advantages, such as reduced waiting times for results, ease of use, and improved communication with parents [26]. Nonetheless, the benefit of CRP POCT for children in primary care has been questioned [13]. Previous meta-analyses have revealed that CRP POCT could reduce antibiotic prescribing for children if combined with clinical guidance [14,30]. A more remarkable effect on antibiotic prescribing was observed in low- and middle-income countries [16]. CRP POCT in primary care is also valuable for ruling out serious bacterial infections [31].

During the study period, GPs were not restricted in performing CRP POCT. However, in the course of the educational session, they were instructed that measuring this marker would be particularly useful for patients with respiratory tract infections and fever, especially when a severe bacterial infection was suspected. Although CRP POCT is not recommended for patients with sore throat, common cold or acute otitis media [26], we observed a high level of testing in the combined intervention group for these ailments. This finding has also been recently reported in studies conducted in Sweden [32,33]. Extensive use of CRP POCT is not recommended and may be associated with unnecessary antibiotic prescribing due to difficulties with the interpretation of intermediate values [26,34]. Having said that CRP POCT could support communication between GPs and the parents of patients in case where there is parental insistence on antibiotics being prescribed [26].

For lower respiratory infections, CRP POCT is recommended for adults on the basis that it has reduced antibiotic prescribing from 53% to 31% [30,35,36]; For children, the use of CRP POCT has resulted in a significant reduction in antibiotic prescribing in low- and middle- income countries [16,37], however, the effect in lower prescribing countries has been reported to be less evident [22,38]. For children younger than 5 years old – the predominant age group in our study – lower respiratory infections are mostly of viral aetiology. Recent guidelines developed by an interdisciplinary working group in Finland do not recommend routine CRP testing for lower respiratory infections in children in primary care [39]. In our study, GPs in the combined intervention group used CRP POCT extensively for acute bronchitis (79.3%) and pneumonia (69.2%). GPs in the usual care group, where CRP POCT was unavailable, still tested 44.0% of pneumonia patients; however, only 14.2% of acute bronchitis patients were tested.

As expected, most of the tested patients in our study had a low CRP level (below 20 mg/L for 78.3% of patients). We observed a higher median CRP value for patients with an antibiotic prescription (24.0 mg/L) compared to patients without an antibiotic prescription (2.9 mg/L). In comparison, the median CRP value for patients with an antibiotic prescription has previously been reported as 23 mg/L for high-prescribing GPs and 45 mg/L for low-prescribing GPs [34]. A threshold of CRP <5 mg/L in primary care [31] and CRP <20 mg/L in hospital emergency departments [40] has been recommended as a safe marker for ruling out serious infection. Patients with serious infections tend to have higher CRP values (median CRP 21 mg/L) compared to patients without a serious infection (median CRP 10 mg/L) [13]. Nevertheless, there is a substantial range of CRP values that correlate with bacterial as well as viral infections and so a definitive decision cannot be reached from only a single CRP measurement. In our study, antibiotic prescribing increased with rising CRP levels, particularly when CRP exceeded 20 mg/L. This finding is in line with data from a study conducted in Norway [41]. Although, strict cut-off values are not well determined [13], a group of experts did recently recommend avoiding antibiotic prescribing if the CRP level is <20 mg/L and clinical assessment rules out a severe infection. Instead, GPs should provide safety netting and consider a second consultation or repeat CRP measurement if the condition worsens. If the CRP level rises above 75 mg/L, then it is strongly advised to start antibiotic treatment [1].

Strep A test was not heavily utilised in all the study groups for acute pharyngotonsillitis (19.3% combined intervention group, 29.1% usual care group, 12.9% long-term education group). Instead, CRP POCT was used for most of the pharyngotonsillitis patients in the combined intervention group (70.6%), which is contrary to guidelines and may be associated with higher antibiotic prescribing compared to no CRP testing [32]. Antibiotic prescribing differed greatly between patients with a positive and ones with a negative Strep A. Most of the pharyngotonsillitis patients with a positive Strep A test received an antibiotic prescription (97.2%), which is in line with sore throat guidelines [42] and available quality indicators for primary care [6]. However, a negative Strep A test often did not convince GPs to withhold antibiotics as 40.7% of pharyngotonsillitis patients with a negative Strep A test also received an antibiotic prescription. This level of prescribing is more than that reported in Sweden, where even high-prescribing GPs prescribed antibiotics for only 35% of patients with a negative Strep A [34].

The level of influenza testing was similar in all three study groups. Influenza RADT (rapid antigen detection test) has been recommended during influenza season as influenza-positive children may benefit from antiviral treatment [39]. Futhermore, conformation of influenza by RADT may reduce unnecessary antibiotic prescribing [43].

Chest X-ray was used to a similar extent for bronchitis across all three study groups; however, for pneumonia, its utilisation was significantly higher in the control group compared to the combined intervention group (56.0% vs. 28.8%). Recent evidence indicates that most children with lower respiratory tract infections do not benefit from chest X-rays, and their use should be avoided in children with a low predicted risk of pneumonia [43]. Furthermore, patients undergoing X-ray tend to receive antibiotics more frequently [38,43].

Only a small number of patients in our study had non-respiratory infections. Among patients with urinary tract infections, CRP testing was performed frequently in the combined intervention group (70.0%), whereas urinalysis was performed relatively infrequently (45.0%). However, there are no current recommendations supporting CRP POCT for these patients.

## Conclusion

CRP POCT in combination with GP education significantly increased the availability and use of diagnostic tests prior to antibiotic prescribing for children with acute infections in primary care. The improvements were most evident in practices in rural regions and with limited access to laboratories. Further improvements in more rational testing and interpretation of test results are also essential to help guide appropriate antibiotic prescribing.

## Data Availability

The original contributions presented in the study are included in the article/supplementary material. Further inquiries can be directed to the corresponding author.
